# Cerebrospinal Fluid (CSF) Analysis and Interpretation in Neurocritical Care for Acute Neurological Conditions

**DOI:** 10.5005/jp-journals-10071-23187

**Published:** 2019-06

**Authors:** Ajay Prasad Hrishi, Manikandan Sethuraman

**Affiliations:** 1,2 Department of Anaesthesiology, Sree Chitra Tirunal Institute for Medical Sciences and Technology, Thiruvananthapuram, Kerala, India

**Keywords:** Acute neurological condition, Cerebrospinal fluid analysis, Demyelination: trauma, Meningitis, SAH

## Abstract

Cerebrospinal fluid (CSF) is a clear fluid circulating in the intracranial and spinal compartments. Under normal conditions, the composition of CSF remains constant. However, in various neurological disease especially in acute conditions, the composition, quantity and its pressure can be altered. By measuring the levels of various CSF components using relevant techniques, diagnosis, severity and prognostication of neurological conditions like infections, subarachnoid hemorrhage, demyelinating conditions, tumor like conditions, etc. can be done. In this review, alterations in CSF components and its relevance to the emergency care physician to help in the management of patients are enumerated.

**How to cite this article:**

Hrishi AP, Sethuraman M. Cerebrospinal Fluid (CSF) Analysis and Interpretation in Neurocritical Care for Acute Neurological Conditions. Indian J Crit Care Med 2019;23(Suppl 2):S115–S119.

## INTRODUCTION

Cerebrospinal fluid is a clear fluid which is formed as a ultra filtrate of plasma. CSF is present in both the intracranial and spinal compartments. It is continuously being secreted by the choroid plexus at a constant rate inside the ventricles of the brain and circulates in the subarachnoid space of the brain and spinal cord through CSF pathways. The total volume of CSF in the adult is approximately 140 mL. CSF is produced at a rate of 0.2–0.7 mL per minute or 500–700 mL per day.^1^ The main function of the CSF is to reduce buoyancy of the brain. It also supplies nutrients as well as helps in removal of various substances like amino acids, neurotransmitters, metabolic byproducts and cells.

The composition of the CSF and its pressure is maintained relatively constant by various mechanisms. However in disease conditions the composition and pressure of CSF can be altered. Hence analysis of CSF by various methods will help in diagnosis as well as prognostication and response to therapy. CSF analysis is particularly useful in various acute neurological conditions and helps in rapid diagnosis of the conditions and initiate therapeutic measures. CSF analysis usually consists of opening pressure measurement, biochemical analysis, cytology, biomarkers assay, and microbiological evaluation.^2^ In some clinical conditions, lumbar puncture and drainage of can be a therapeutic measure also.^3^

### Composition of Normal CSF

The normal composition and pressure (lumbar) of CSF consists of the following^1^ (Table 1);

### Sample collection

It is important to time the lumbar puncture (LP) to get maximum diagnostic yield (Tables 2 and 3). Moreover if the patient is having coagulopathy or anticoagulants/antiplatelet drugs, LP must be done depending on the duration of drug action and guidelines for LP by (American Society of Regional Anesthesia- ASRA) can be followed.^4^ In patients with seizures suspected to be due to certain metabolic illness, a fasting LP is taken for CSF analysis. In patients whose sensorium obtunded, CT scan of brain must be done to rule of signs of raised ICP, mass lesions due to risk of brain herniation due to LP. Sedation may be required in uncooperative patients and children, and fasting guidelines must be ensured to prevent aspiration of gastric contents.

**Table 1:** Normal composition of CSF

	*Normal range*
Color	Clear
Specific gravity/pH	1.006–1.007/7.4
Opening pressure	50–200 mm H_2_O
RBCs count	Nil
WBC count	0–5 (upto 30 in neonates)
WBC types	Lymphocytes
CSF Proteins	15–40 mg/dL
CSF lactate	1–3 mmol/ L
CSF glucose	50–80 mg/dL (two thirds of blood glucose
Microbial examination	No microorganism

LP can be performed either in lateral or sitting posture. Usually under sterile precautions, 22–24 G spinal needle is inserted after identifying the lumbar L2–3 or L3–4 space and local infiltration. Once the spinal subarachnoid space is identified using loss of resistance, controlled removal of stylet is done to prevent excessive drainage of CSF. A manometer can be connected if CSF opening pressure measurement is planned. Color of CSF is noted and if blood stained due to traumatic puncture, it may be needed to wait for the blood to be cleared before samples are collected. Samples are usually collected in three to four test tubes each of 3–5 mL CSF for analysis. Samples are transported to lab in biohazard bag without refrigeration.

**Table 2:** Indications for lumbar puncture

*Diagnostic*
CNS infections
Autoimmune CNS diseases like Guilliain Barrie syndrome
CNS vasculitis
CT negative subarachnoid hemorrhage
Malignant cells in metastasis
For injection of dye like fluorescin to identify site of CSF leaks
*Therapeutic*
Benign intracranial hypertension
Acute communicating hydrocephalus
Cryptococcal meningitis in HIV infections
For CSF leaks
*Delivery of intrathecal drugs*
Delivery of antibiotics
Delivery of antineoplastic drugs

**Table 3:** Contraindications for LP

Relative contraindications Platelet count of less than 20000–40000/cu mm Thienopyridines therapy
Absolute contraindications Non-communicating obstructive hydrocephalus Uncorrected bleeding diathesis Anticoagulant therapy (timing of LP depends on the stopping of anticoagulant drug) Platelet count less than 20000/ cu mm Spinal canal stenosis or spinal cord compression above level of puncture Local skin infections

### Approach to CSF analysis in Acute Brain Conditions

#### CSF in Subarachnoid Hemorrhage (SAH)

Subarachnoid Hemorrhage (SAH) is one of the acute neurological condition needing intensive care admission of the patient. The causes for SAH can be due to rupture of intracranial aneurysms, arteriovenous malformations (AVM), vasculitis, traumatic or idiopathic. Most commonly patients present with acute onset severe headache increasing intensity within one hour of onset and often describing as worst headache of their life. In severe cases patients can present with loss of consciousness (LOC), seizures, cranial nerve palsies and various patterns of motor deficits. It is important to recognize the condition immediately as rebleed in case of aneurysmal cause can be catastrophic. Since headache is a common manifestation of intracranial diseases high index of suspicion is needed. In a recent study, Perry et al have found that following patient factors must lead to suspicion of SAH; age ≥40, witnessed loss of consciousness, complaint of neck pain or stiffness, onset with exertion, arrival by ambulance, vomiting, diastolic blood ≥100 mm Hg or systolic blood ≥160 mm Hg.^5^

Before the advent of CT scanners, LP was the main diagnostic tool for SAH Presently plain CT scan is the initial diagnostic modality. However at times despite clinical suspicion, CT scan may be negative for SAH. CT is considered to be highly sensitive upto 5 days after the SAH. Lumbar puncture is a useful diagnostic test in these cases and is usually done 12 hours after onset of symptoms. LP is indicated if there is high clinical suspicion of SAH, but negative CT. LP is usually done after few hours. The presence of RBCs (More than 1000 cells/ cu mm) or xanthochromia (Level 2 evidence recommendation B) confirms diagnosis of SAH.^6,7^

However traumatic LP can complicate the RBC leading to false positive. To distinguish traumatic tap Vs SAH, CSF is collected in three test tubes and by counting the red cells in the consecutive tubes. A decrease in the red cells would usually indicate a traumatic tap. Another method is to centrifuge the CSF and look for xanthochromia of the supernatant fluid. Spectrophotometry is recommended for xanthochromia detection. Xanthochromia could be seen as early as 12 hours which is due to lysis of RBCs in CSF and release of pigments like oxyhemoglobin, methemoglobin and bilirubin.^8^ However for the diagnosis of rebleeding of SAH, LP is not useful due to presence of pigments till 3 weeks of first SAH.

### CSF Analysis in Meningitis/ Meningoencephalitis

Patients with suspected meningitis is one of the major indication for LP and CSF study. Meningitis can be community acquired or hospital acquired and caused by various micro organisms ranging from bacteria, virus, fungus, protozoa, etc.^9,10^ Aseptic meningitis is a condition that needs to be distinguished from other forms of meningitis that need a CSF analysis.^11^ Presentation of meningitis varies from acute debilitating illness or chronic symptoms as in tuberculosis. Patients suspected to have acute meningitis usually present with altered consciousness, fever and neck stiffness. The classic triad is seen only in 46% of patients. In others one or two of the signs of triad may be present. In addition patients can present with nausea, vomiting, headache and photophobia. In patients with meningoencephalitis additional clinical signs at presentation include altered sensorium, confusion, behavioural changes, seizures, focal neurological deficits.

Once there is high index of suspicion of meningitis usually a single dose of antibiotic is started empirically after taking blood cultures and without waiting for confirmation by LP. A CT scan may be indicated before LP if the patient has suspicion of raised ICP or mass lesion like cerebral edema, or herniation. However CSF analysis is the gold standard for diagnosis of meningitis as well as to identify its etiology (Table 4).

During LP the opening pressure of CSF, samples for routine biochemistry, cell counts are undertaken. In addition CSF samples are used for Gram Stains, microbial culture, antibodies against antigens using polymerase chain reaction (PCR), and immuno histochemistry analysis.

### CSF Analysis in Acute Demyelinating/Autoimmune Diseases

Demyelinating diseases are a spectrum of diseases where the patient may present with acute neurological condition to ED. CSF analysis can be very useful in diagnosis of certain diseases and can aid in progress of disease and prognostication following therapy.

The demyelinating diseases include multiple sclerosis, acute disseminated encephalomyelitis, and neuromyelitis optica (NMO).^12,13^ In addition to above diseases, various autoimmune diseases like Guilliain Barrie syndrome, Transverse myelitis, etc can also cause varying degrees of demyelination and degeneration.^14,15^ These conditions may result in acute emergencies because of severe inflammatory destruction of CNS tissues or complications thereof.

**Table 4:** CSF analysis in various types of meningitis

*CSF analysis*	*Bacterial*	*Tuberculosis*	*Viral*	*Fungal*	*Aseptic*
Pressure	Increased	Increased	Normal to elevated	Normal to mild increase	Increased
Color	Turbid	Turbid	Clear	Clear	Clear to turbid
Glucose	< 40 mg%	Low	Normal to mild less	Low to normal	Low
Proteins	Elevated	Greatly elevated	Normal to mild elevation	Normal to mild increase	Elevated
Lactate	Elevated (> 6 mmol/L)	Elevated	0–6 mmol/L	Normal	Normal
RBCs	Elevated	Elevated	Normal	Normal	Elevated
WBCs	10-2000/ cu mm	Elevated, but < 500	>100/ cu mm	10–50/. cu mm	Mildly elevated
WBC types	Neutrophils	Lymphocytes	Lymphocytes	Lymphocytes	Neutrophils
Gram stain	Positive	Acid fast bacilli	Negative	Negative India ink for spores/fungi	Negative
Microbial Culture	Positive	Positive (yield is high in early stages)	Negative	Positive	Negative
Biomarkers	Elevated C-reactive proteins	Antibodies in CSF (detection of anti-M37Ra, anti-antigen 5, and anti-M37Rv)			
		Elevated CSF procalcitonin, Adenosine deaminase	Low CRP and adenosine deaminase		Seen following neurosurgery or antibiotic use
PCR test		Help in identification of organisms even after antibiotics are started	Helps in identification of the organisms		

The pathological findings in these group of diseases includes the presence of focal collections of lymphocytes and monocytes, with varying degrees of demyelination, axonal injury, and astroglial and microglial activation. In addition compliment factors deposition, vasculitis of the blood vessel can be seen. The pathology usually follow a post infectious, or post vaccination or systemic inflammatory response. Leading to autoimmune or inflammatory response to CNS components. Most of these conditions are responsive to therapy and hence early diagnosis and treatment leads to better outcomes for the patient. CSF analysis give valuable information regarding the diagnosis, progression or relapse of the disease as well as effect of treatment (Table 5).

### Brain and Spinal Cord Neoplasms

Neoplasms of CNS in which CSF analysis are helpful is usually due to primary CNS lymphoma or secondary leptomeningeal deposits. Patients can present acutely with features of raised intracranial pressure due to mass effect, cerebral edema or herniations. Radiological investigations will help in making the diagnosis. However at times, it may be difficult to distinguish between secondaries and primary tutors or infections like abscess. A CSF analysis may help.^16^ After the initial CT scan brain or MRI, centrifuged CSF can be used for presence or absence of malignant cells and various tumor markers like colon, lung, prostate etc.

### CSF Analysis in Spinal Cord Diseases

#### Guillain Barre Syndrome (GBS)

- *Albumino-Cytologic Dissociation:* The classic immunologic alteration of CSF is high CSF protein with normal cell count (albuminocytologic dissociation). Pleocytosis may rarely also occur.
- *Neurofilament Light (NFL) Protein Assay:* The NFL level correlated with disability in the acute phase of GBS and was strongly predictive of persistent disability. NFL levels were dependent on age in the control group, independent of gender, and correlated with the albuminocytologic dissociation which is indicator of the integrity of the blood-brain barrier in patients.^17^

### Amyotrophic Lateral Sclerosis (ALS)

Micro RNA (miR), miR-218 depletion in ALS spinal cord tissue is likely due to motor neurons loss because miR-218 levels are mostly maintained within the surviving motor neurons. miR-218 release is related to motor neurons dysfunction and death and occurs in both animal and human models of motor neurons loss and disease.^18^

### Spinal Cord Ependymoma

In cases where the tumor is not surrounded by parenchyma or isolated by arachnoid membranes, tumor-derived cell free DNA (cfDNA) can be detected. Tumor-derived cfDNA is found in the CSF of six of seven tumor patients.^19^

### Infectious vs Non-infectious Spine Pathology

It is important to differentiate infections vs non-infectious spinal cord pathology and CSF analysis will help in diagnosing these conditions.^20^

Each cytokine has been identified as potentially useful in the diagnostic discrimination of CNS diseases has been discussed in the literature and is associated with various aspects of these disease processes. The analysis following cytokines plays an important role in the diagnosis of spine pathologies: macrophage derived chemokine (MDC/CCL22), granulocyte macrophage colony-stimulating factor GRO/CXCL10, Interleukins (IL-12p70, IP-10/CXCL10), Platelet-derived growth factor AA (PDGF-AA).

**Table 5:** CSF changes in acute demyelinating/inflammatory diseases

*Condition*	*Clinical features*	*CSF findings*
Transverse myelitis	Bilateral (not necessarily symmetric) sensorimotor and autonomic spinal cord dysfunction Clearly defined sensory level Hyperreflexia, babinski positive	Signs of inflammation (pleocytosis, elevated protein concentration, oligoclonal bands, or elevated IgG index) Elevated CSF IL-6 PCR negative of infections. CSF sugar, pressure usually normal.
Multiple sclerosis (different types)	Loss of sensation Muscle weakness Visual loss Incoordination, cognitive impairment Fatigue, pain Bladder and bowel disturbance	Pleocytosis (5–50 cells / cu mm; lymphocytes) Elevated protein OCB may be present. (highly diagnostic) Ig G index (increase CSF IgG compared to serum IgG levels)
Neuromyelitis optica	A severe transverse myelitis. An acute unilateral or bilateral optic neuropathy. No other clinical involvement.	Non specific Pleocytosis (5–50 cells/ cu mm; lymphocytes) Elevated protein OCB may be present. (most cases) Normal glucose levels
ADEM	Fever, meningeal signs, and acute encephalopathy The level of consciousness ranges from lethargy to frank coma. Maximum progression 4–7 days Common in children MRI diagnostic	Pleocytosis (5–50 cells/cu mm) and/or increased protein concentration May be normal CSF non diagnostic. Presence of oligoclonal band (OCB) favours diagnosis of MS
Guillain Barrie syndrome	Acute progressive weakness, areflexia, symmetry Post infection Autonomic dysfunction Cranial nerve involvement Mild sensory signs	Normal CSF cell count. Elevated CSF protein levels Cyto-albuminergic disassociation

**Flowchart 1:**
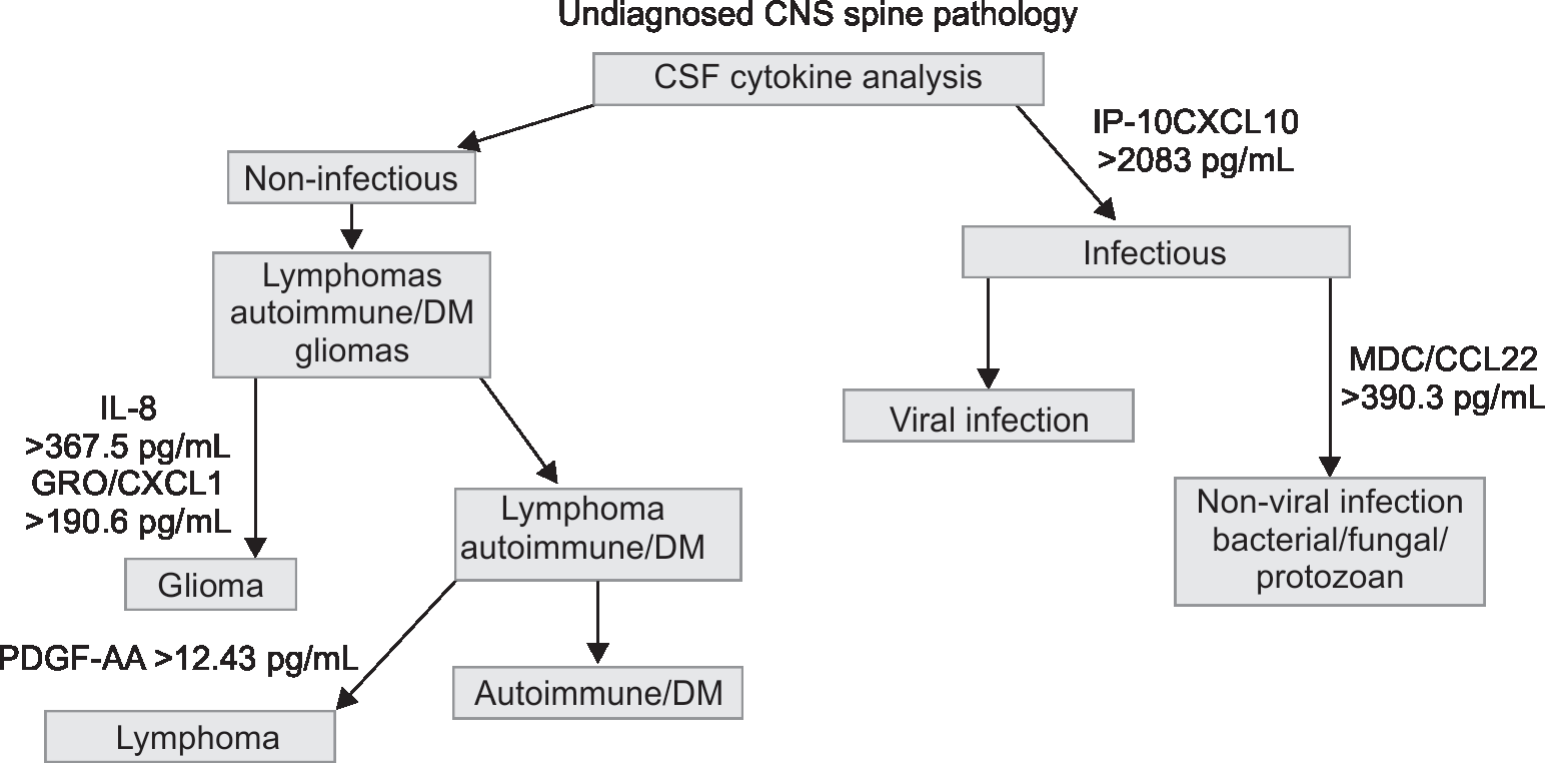
Algorithmic approach to a undiagnosed CNS/spinepathology [MDC/CCL22, macrophage derived chemokine; GRO/CXCL10, granulocyte macrophage colony-stimulating factor; IL-12p70, IP-10/CXCL10, interleukins; PDGF-AA, platelet-derived growth factor AA]

Determination of IP-10/CXCL10 CSF levels provides an important early branch point in the diagnostic algorithm. It is important to differentiate infections vs non-infectious spinal cord pathology and CSF analysis will help in diagnosing these conditions^20^ (Flowchart 1). Elevated IP-10/CXCL10 levels rapidly identify a probable infection. Further sub-classification of an infectious case can done by assessing the MDC/CCL22 levels. High MDC/CCL22 levels suggest a non-viral infectious agent rather than a viral pathogen. In the non-infectious arm of the algorithm i.e;low IP-10/CXCL10 levels), elevated levels of IL-8 and GRO/CXCL1 favor a diffuse glioma. Autoimmune/Demyelination(DM) and CNS B-cell lymphoma can be further delineated by using PDGF-AA.

### CSF Analysis and Biomarkers in Spinal Cord Injury

Spinal cord injury can cause elevation of various biomarkers. These included micro RNA(mRNA), neuron specific enolase (NSE), neurofilaments (NF), glial fibrillary proteins, Interlukein (IL6) and cleaved Tau proteins. These represent different components of damage like neurons, axons, glial cells, vascular injury, food spinal cord barrier. Analysis of various biomarkers in the CSF in patients who has sustained injury to spinal cord. Can help in assessing the severity of injury as well as in prognostication of patients.^21-23^

### CONCLUSION

CSF analysis is a very useful investigations in patients presenting with acute neurological conditions. While it is mandatory for acute meningitis to identify the microbial organisms, it will help as a supporting investigation to establish diagnosis, prognosticate and to assess the effect of various therapies to treat the patient conditions. CSF analysis has evolved over years form basic analysis to assessment of various biomarkers to aid the clinician. Hence it is very imperative for the physician to understand the indications for these investigations as these are not widely available and expensive.

## References

[1] Seehusen DA,, Reeves MM,, Fomin DA. (2003). Cerebrospinal fluid analysis.. Am Fam Physician..

[2] Hepnar D,, Adam P,, Žáková H,, Krušina M,, Kalvach P,, Kasík J, (2019;). Recommendations for cerebrospinal fluid analysis.. Folia Microbiol (Praha)..

[3] Costerus JM,, Brouwer MC,, van de Beek D. (2018;). Technological advances and changing indications for lumbar puncture in neurological disorders.. Lancet Neurol..

[4] Horlocker TT,, Wedel DJ,, Rowlingson JC,, Enneking FK,, Kopp SL,, Benzon HT, (2010;). Regional anesthesia in the patient receiving antithrombotic or thrombolytic therapy: American Society of Regional Anesthesia and Pain Medicine Evidence-Based Guidelines (Third Edition).. Reg Anesth Pain Med..

[5] Perry JJ,, Stiell IG,, Sivilotti ML,, Bullard MJ,, Lee JS,, Eisenhauer M, (2010). High risk clinicalcharacteristics for subarachnoid haemorrhage in patients with acute headache:prospective cohort study.. BMJ..

[6] Vivancos J,, Gilo F,, Frutos R,, Maestre J,, García-Pastor A,, Quintana F, (2014;). Guía de actuación clínica en la hemorragia subaracnoidea. Sistemática diagnóstica y tratamiento.. Neurología..

[7] Carpenter CR,, Hussain AM,, Ward MJ,, Zipfel GJ,, Fowler S,, Pines JM, (2016;). Spontaneous Subarachnoid Hemorrhage: A Systematic Review and Meta-analysisDescribing the Diagnostic Accuracy of History, Physical Examination, Imaging, and Lumbar Puncture With an Exploration of Test Thresholds.. Acad Emerg Med..

[8] M Vermeulen,, Gijn J van. (1990;). The diagnosis of subarachnoid haemorrhage.. J Neurol Neurosurg and Psychiatry..

[9] Griffiths MJ,, McGill F,, Solomon T. (2018;). Management of acute meningitis.. Clin Med(Lond)..

[10] Tunkel AR,, Hasbun R,, Bhimraj A,, Byers K,, Kaplan SL,, Michael Scheld W, (2017;). Infectious Diseases Society of America's Clinical Practice Guidelines for Healthcare-Associated Ventriculitis and Meningitis.. Clin Infect Dis..

[11] Kumar R. (2005;). Aseptic Meningitis: Diagnosis and Management.. Indian J Pediatr.

[12] Freedman MS,, Thompson EJ,, Deisenhammer F,, Giovannoni G,, Grimsley G,, Keir G, (2005;). Recommended standard of cerebrospinal fluid analysis in the diagnosis of multiple sclerosis: a consensus statement.. Arch Neurol..

[13] Matas SL,, Glehn Fv,, Fernandes GB,, Soares CA. (2013;). Cerebrospinal fluid analysis in the context of CNS demyelinating diseases.. Arq Neuropsiquiatr..

[14] Willison HJ,, Jacobs BC,, van Doorn PA. (2016;). Guillain-Barré syndrome.. Lancet..

[15] Rodríguez Y,, Rojas M,, Pacheco Y,, Acosta-Ampudia Y,, Ramírez-Santana C,, Monsalve DM, (2018;). Guillain-Barré syndrome, transverse myelitis and infectious diseases.. Cell Mol Immunol..

[16] Ballester LY,, Lu G,, Zorofchian S,, Vantaku V,, Putluri V,, Yan Y, (2018;). Analysis of cerebrospinal fluid metabolites in patients with primary or metastatic centralnervous system tumors.. Acta Neuropathol Commun..

[17] Axelsson M,, Sjögren M,, Andersen O,, Blennow K,, Zetterberg H,, Lycke J. (2018;). Neurofilament light protein levels in cerebrospinal fluid predict long-term disability of Guillain-Barré syndrome: A pilot study.. Acta Neurol Scand..

[18] Claudia R,, Marzocchi Carlotta,, S Stefania B. (2018;). Micro RNAs as Biomarkers in Amyotrophic lateral sclerosis.. Cells.

[19] Pan W,, Gu W,, Nagpal S,, Gephart MH,, Quake SR. (2015;). Brain tumor mutations detected in cerebral spinal fluid.. Clin Chem..

[20] Fortuna D,, Hooper DC,, Roberts AL,, Harshyne LA,, Nagurney M,, Curtis MT. (2018;). Potential role of CSF cytokine profiles in discriminating infectious from non-infectious CNS disorders.. PLoS ONE.

[21] Seth T,, Rihab G,, Casey PS,, Femke S,, Sunita S,, Stephanie F, (2019.). MicroRNA biomarkers in cerebrospinal fluid and serum reflect injury severity in human acute traumatic spinal cord injury.. J Neurotrauma..

[22] Kwon BK,, Streijger F,, Fallah N,, Noonan VK,, Bélanger LM,, Ritchie L, (2017;). Cerebrospinal fluid biomarkers to stratify injury severity and predict outcome in human traumatic spinal cord injury.. J Neurotr..

[23] Yokobori S,, Zhang Z,, Moghieb A,, Mondello S,, Gajavelli S,, Dietrich WD, (2015;). Acute diagnostic biomarkers for spinal cord injury: review of the literature and preliminary research report.. World Neurosurg..

